# Supplementary material for: Job Demands, Work Functioning and Mental Health in Dutch Nursing Home Staff during the COVID-19 Outbreak: A Cross-Sectional Multilevel Study
Source: Int J Environ Res Public Health. 2022 Apr 6;19(7):4379. doi: 10.3390/ijerph19074379 (PMC8998775; doi:10.3390/ijerph19074379)
Supplement: Supplementary file 1 [file ijerph-19-04379-s001.zip › ijerph-1638084-supplementary.pdf]

Supplementary Table S1. Sensitivity analysis care staff (*n* = 1148). Differences between nursing home staff with and without COVID contact,or COVID infection and across different levels of COVID worries in job demands. Reference group: COVID contact (no), COVID infection (no), COVID worries (always).

	Job Demands								
	Quantitative Demands			Cognitive Demands			Emotional Demands		
	**B**	***p***	**95% CI**	**B**	***p***	**95% CI**	**B**	***p***	**95% CI**
Intercept	56.16 [1]		52.79 to 59.52	64.09		63.10 to 65.08	64.49 [1]		62.84 to 66.15
COVID contact (no)	0			0			0		
COVID contact (yes)	2.24 [1]	0.062	−0.11 to 4.60	1.42	0.168	−0.60 to 3.45	1.98 [1]	0.056	−0.05 to 4.01
COVID infection (no)	0			0			0		
COVID infection (yes)	0.40 [2]	0.879	−5.53 to 6.33	−0.78	0.672	−4.41 to 2.84	3.44 [1]	0.060	−0.15 to 7.03
COVID worries									
always (4)	0			0			0		
often (3)	−7.77 [1]	<0.001	−12.03 to −3.52	−10.53	<0.001	−14.69 to 6.36	−11.36 [1]	<0.001	−15.36 to 7.36
sometimes (2)	−13.49 [1]	<0.001	−17.71 to −9.26	−14.77	<0.001	−18.91 to 10.63	−17.81 [1]	<0.001	−21.78 to 13.84
rarely (1)	−17.13 [1]	<0.001	−22.62 to −11.65	−16.96	<0.001	−22.31 to 11.61	−22.90 [1]	<0.001	−28.05 to 17.75
never (0)	−26.20 [1]	<0.001	−35.54 to −16.87	−17.67	<0.001	−26.70 to 8.64	−20.92 [1]	<0.001	−29.69 to 12.16

[1] included random intercepts, [2] included random intercepts and slopes, % missing (range): QD: 6–7%, CD: 6–7%, ED: 6–7%.

Supplementary Table S2. Sensitivity analysis care staff (n = 1148). Differences between nursing home staff with and without COVID contact or COVID infection and across different levels of COVID worries in work functioning and mental health. Reference group: COVID contact (no), COVID infection (no), COVID worries (always).

	Work Functioning and Mental Health								
	Work Functioning			Depressive Symptoms			Burnout		
	**B**	***p***	**95% CI**	**B**	***p***	**95% CI**	**B**	***p***	**95% CI**
Intercept	77.27		76.08 to 78.45	4.27 [1]		3.74–4.81	2.46 [1]		2.34 to 2.58
COVID contact (no)	0			0			0		
COVID contact (yes)	−0.15	0.900	−2.56 to 2.25	0.30 [1]	0.251	−0.21 to 0.81	0.004 [1]	0.932	−0.09 to 0.10
COVID infection (no)	0			0			0		
COVID infection (yes)	−4.48	0.046	−(8.87 to −0.08)	1.73 [1]	<0.001	0.84 to 2.61	0.32 [1]	<0.001	0.15 to 0.49
COVID worries									
always (4)	0			0			0		
often (3)	7.32	0.005	2.23 to 12.42	3.19 [1]	<0.001	−4.22 to −2.17	−0.44 [1]	<0.001	−0.63 to −0.24
sometimes (2)	12.87	<0.001	7.80 to 17.94	−4.94 [1]	<0.001	−5.96 to −3.92	−0.72 [1]	<0.001	−0.91 to −0.52
rarely (1)	15.53	<0.001	9.07 to 21.99	−4.90 [1]	<0.001	−6.20 to −3.59	−0.86 [1]	<0.001	−1.11 to −0.61
never (0)	12.54	0.025	1.62 to 23.46	−5.18 [1]	<0.001	−7.36 to −2.99	−0.69 [1]	0.001	−1.10 to −0.28

[1] included random intercepts, % missing (range): WF: 25–26%, DS: 19–20%, BO: 11–12%.
